# Geographical Origin Identification of Chinese Red Jujube Using Near-Infrared Spectroscopy and Adaboost-CLDA

**DOI:** 10.3390/foods14050803

**Published:** 2025-02-26

**Authors:** Xiaohong Wu, Ziteng Yang, Yonglan Yang, Bin Wu, Jun Sun

**Affiliations:** 1 School of Electrical and Information Engineering, Jiangsu University, Zhenjiang 212013, China; sun2000jun@ujs.edu.cn; 2 High-Tech Key Laboratory of Agricultural Equipment and Intelligence of Jiangsu Province, Jiangsu University, Zhenjiang 212013, China; 3 Mengxi Honors College, Jiangsu University, Zhenjiang 212013, China; 3210602036@stmail.ujs.edu.cn; 4 School of Energy and Power and Engineering, Jiangsu University, Zhenjiang 212013, China; 3210212090@stmail.ujs.edu; 5 Department of Information Engineering, Chuzhou Polytechnic, Chuzhou 239000, China; 6 School of Computer Science and Engineering, Southeast University, Nanjing 211102, China

**Keywords:** red jujube, near-infrared spectroscopy, feature extraction, geographical origin

## Abstract

Red jujube is a nutritious food, known as the “king of all fruits”. The quality of Chinese red jujube is closely associated with its place of origin. To classify Chinese red jujube more correctly, based on the combination of adaptive boosting (Adaboost) and common vectors linear discriminant analysis (CLDA), Adaboost-CLDA was proposed to classify the near-infrared (NIR) spectra of red jujube samples. In the study, the NIR-M-R2 spectrometer was employed to scan red jujube from four different origins to acquire their NIR spectra. Savitzky–Golay filtering was used to preprocess the spectra. CLDA can effectively address the “small sample size” problem, and Adaboost-CLDA can achieve an extremely high classification accuracy rate; thus, Adaboost-CLDA was performed for feature extraction from the NIR spectra. Finally, K-nearest neighbor (KNN) and Bayes served as the classifiers for the identification of red jujube samples. Experiments indicated that Adaboost-CLDA achieved the highest identification accuracy in this identification system for red jujube compared with other feature extraction algorithms. This demonstrates that the combination of Adaboost-CLDA and NIR spectroscopy significantly enhances the classification accuracy, providing an effective method for identifying the geographical origin of Chinese red jujube.

## 1. Introduction

Chinese red jujube is a kind of dried fruit with edible, medicinal, and healthcare functions [1,2,3]. It contains protein, sugars, organic acids, vitamin A, vitamin C, and other rich nutrients, and it has some healthcare effects such as replenishing the spleen and stomach qi and nourishing blood for tranquillization. Different soil and climate conditions in various regions can affect the quality and chemical composition of jujubes [4,5], thereby influencing their taste. Investigating the origin of jujube can provide insights into how environmental factors affect jujube [6]. Variations in soil, climate, and cultivation practices among regions may result in unique characteristics, potentially appealing to consumers [7]. Above all, investigating the origin of jujube provides valuable information about its growth environment, quality characteristics, and potential medicinal value. This can be beneficial for producers, consumers, researchers, and government regulatory agencies [6,7,8,9].

Several conventional methods have been used for the classification of jujube, focusing on its geographical and varietal traceability. Physical characteristics such as size, shape, color, and weight, along with nutritional properties like sugar, moisture, protein, and phenolic content, are commonly employed. Wu et al. tried a combination of physical measurements (e.g., calipers for size, colorimeters for color) and nutritional analyses (e.g., HPLC for sugar and phenolics) to differentiate Chinese jujubes [10]. While nutritional profiling achieved higher precision, physical traits were less reliable due to overlaps between varieties. These methods, however, are time-consuming, labor-intensive, and often destructive, making them impractical for large-scale applications. Similarly, Wang et al. performed PCA to classify 15 varieties of Chinese jujube based on volatile profiles, but this approach required expensive instrumentation (e.g., GC–MS) and was limited by its procession of high-dimensional data [11].

Near-infrared (NIR) spectroscopy has become a powerful tool in the food industry, providing non-invasive, real-time monitoring, quality control, classification, and safety assurance [12,13,14,15]. For jujube classification, it detects key nutritional components, such as sugar, moisture, protein, and phenolics, without damaging samples [16,17,18]. Wang et al. demonstrated its high accuracy in detecting insect infestations in jujubes [16], while Guo et al. highlighted its precision in chemical composition analysis when combined with chemometric techniques [18]. A major advancement is the development of portable NIR devices, enabling rapid on-site detection at cultivation sites, processing plants, or markets [19]. These devices enhance efficiency, reduce reliance on lab-based methods, and make NIR more accessible. Furthermore, NIR addresses the limitations of traditional methods by eliminating destructive sampling and minimizing chemical use. Integration with the chemometrics methods such as partial least squares regression (PLSR) and back propagation neural network (BPNN) further enhances accuracy, as shown by Luo et al., who developed reliable online detection models for southern Xinjiang jujubes [17]. These advancements establish NIR as a sustainable and practical solution for jujube classification and traceability.

Due to the high dimensions and redundant data in NIR spectra, there is a need for processing NIR data with feature extraction methods. For example, fuzzy improved linear discriminant analysis (FILDA), Adaboost-ULDA, and fuzzy uncorrelated discriminant transformation (FUDT) have been used to extract discriminant data from NIR spectra of foods [20,21,22]. On the other hand, linear discriminant analysis (LDA)-based combination algorithms, such as discriminant partial least squares (DPLS) with LDA, and successive projections algorithm (SPA) with LDA, were utilized to classify NIR spectra of corn and camellia, respectively [23,24]. Discriminant vectors in the null space of $S_{W}$ were computed to derive discriminative common vectors for classification, and then common vectors linear discriminant analysis (CLDA) was proposed for solving the small sample size problem [25]. Inspired by the notion of model fusion, Adaboost integrates multiple relatively weak classifiers to produce a robust ensemble classifier [26]. Leveraging its unique weight-adjustment mechanism and iterative training process, Adaboost has been successfully applied to classify pork storage time with Fourier transform NIR spectroscopy, as well as in other practical applications [21,27].

**Figure 1:**
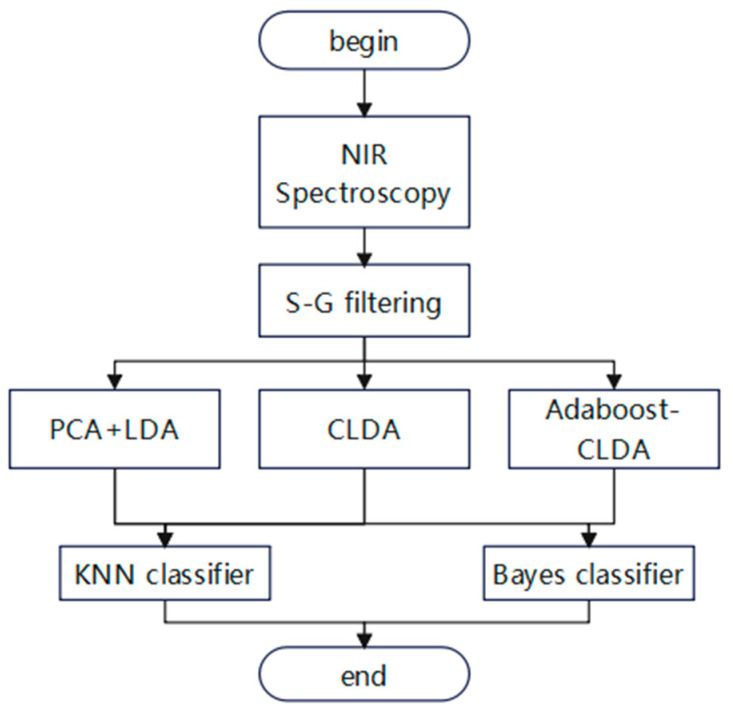
The schematic diagram of the traceability system. PCA, principal component analysis; LDA, linear discriminant analysis; CLDA, common vectors linear discriminant analysis; NIR, near-infrared; S-G, Savitzky–Golay; KNN, K-nearest neighbor.

In this study, the classification system for identification of jujube origins has four parts: data acquisition, data preprocessing, feature extraction, and classification. The NIR-M-R2 spectrometer was used to scan red jujube for NIR spectra, which were pretreated by Savitzky–Golay (SG) filtering. Then, the data features were extracted via PCA + LDA, CLDA, and Adaboost-CLDA, respectively. In the end, K-nearest neighbor (KNN) and Bayes served as classifiers for the identification of red jujube. The schematic diagram of the traceability system for red jujube is presented in Figure 1.

## 2. Materials and Methods

### 2.1. Jujube Sample Preparation

The cultivation areas and varieties of red jujube samples were Dunhuang Junzao (Gansu), Xinzheng Huizao (Henan), Jishan Banzao (Shanxi), and Ruoqiang Huizao (Xinjiang). Experimenters wiped off any dust on the surface of the samples with a soft cloth to guarantee that the appearance of each sample was clean. On the other hand, they carefully selected red jujube samples by rejecting samples with noticeable defects, insect infestation, and other contaminants. A total of 60 samples were collected for each province (i.e., each variety), resulting in a total of 240 samples. Following this, the red jujube samples were partitioned into the training set and test set according to a proportion of 2:1, which meant each variety was subdivided into 40 training samples and 20 test samples.

### 2.2. NIR Acquisition

The NIR-M-R2 spectrometer (Shenzhen Pynect Science and Technology Co. Ltd., Shenzhen, China) was applied to collect the NIR spectra of red jujube samples. This spectrometer operates within a wavelength range of 900–1700 nm, containing an InGaAs detector, a ratio of signal to noise of 6000:1, and a slit size of 1.8 × 0.025 mm. During the data collection, the environmental conditions were controlled, maintaining an experimental temperature of approximately 25 °C and a relative humidity of 50–60%. Before collecting NIR spectra, the spectrometer underwent a preheating period of one hour.

**Figure 2:**
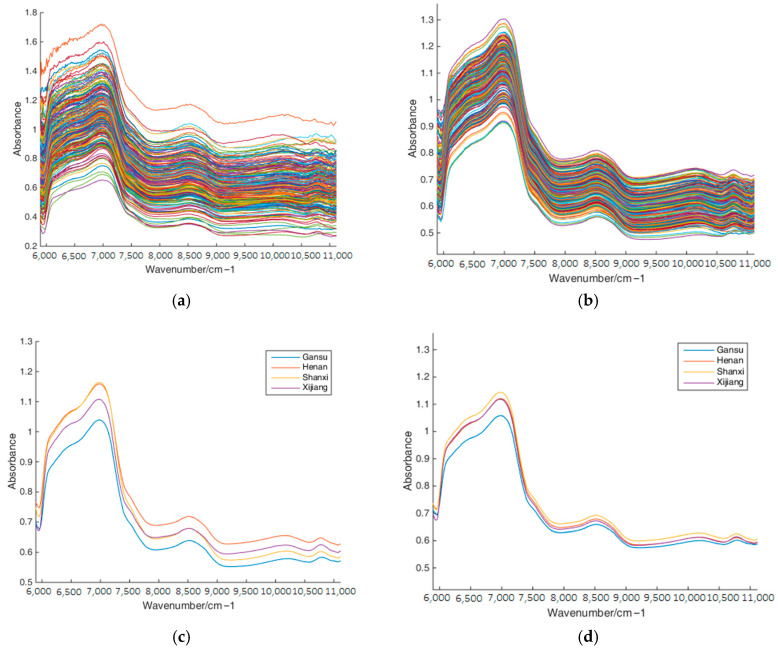
NIR spectra of red jujube. (**a**) The original spectra; (**b**) the preprocessed spectra by SG algorithm; (**c**) tmean spectra; (**d**) the mean spectra preprocessed by SG algorithm.

The NIR spectra represented the 228-dimensional data for each red jujube sample. For consistency and reliability, each red jujube sample underwent three scans along the equator using the spectrometer. The final NIR spectrum of each sample is the average of the three scans for each sample, and they are shown in Figure 2a.

### 2.3. Spectral Data Preprocessing

In order to minimize the impact of noise and some partially redundant information on experimental accuracy, multiple preprocessing algorithms were introduced, including multiplicative scatter correction (MSC), standard normal variable (SNV), and Savitzky–Golay (SG) filtering. SG is effective in eliminating noise from NIR spectra, while MSC and SNV are capable of mitigating the influence of scattering in the spectral data [1].

### 2.4. CLDA

The steps of obtaining the discriminative common vectors using the range space of $S_{W}$ can be summarized as follows:

Consider a training set comprising C classes, each containing N samples. Let $x_{m}^{i}$ represent a d-dimensional column vector indicating the mth sample from the ith class. The total number of samples in the training set is denoted as M=NC. Assuming that d>M−C, in this scenario, $S_{W}$, $S_{B}$, and $S_{T}$ can be defined as follows [25]:

(1) $$S_{W}=\sum_{i=1}^{C}\sum_{m=1}^{N}(x_{m}^{i}-\mu_{i}){\left(x_{m}^{i}-\mu_{i}\right)}^{T}$$

(2) $$S_{B}=\sum_{i=1}^{C}N(\mu_{i}-\mu ){\left(\mu_{i}-\mu \right)}^{T}$$

and

(3) $$S_{T}=\sum_{i=1}^{C}\sum_{m=1}^{N}(x_{m}^{i}-\mu ){\left(x_{m}^{i}-\mu \right)}^{T}=S_{W}+S_{B}$$

where μ is the mean of all samples, and $\mu_{i}$ is the mean of samples of the i th class.

Step 1: Calculate the non-zero eigenvalues and their corresponding eigenvectors of $S_{W}$ using the matrix $A^{T}A$, where $S_{W}=A^{T}A$ and A is defined by Equation (4). The form is $Q=[\alpha_{1},\ldots ,\alpha_{r}]$, where r is the rank of $S_{W}$.

(4) $$A=[x_{1}^{1}-\mu_{1},\ldots ,x_{N}^{1}-\mu_{1},x_{1}^{2}-\mu_{2},\ldots ,x_{N}^{C}-\mu_{C}]$$

Step 2: Select a representative sample from each class and project it onto the null space of $S_{W}$ to derive the common vectors as follows [25]:

(5) $$x_{com}^{i}=x_{m}^{i}-QQ^{T}x_{m}^{i},\;m=1,\ldots ,N,\;i=1,\ldots ,C$$

Step 3: Determine the eigenvectors $w_{k}$ of $S_{com}$, associated with the non-zero eigenvalues, utilizing the matrix $A_{com}^{T}A_{com}$, where $S_{com}=A_{com}A_{com}^{T}$ and $A_{com}$ is defined in Equation (5). There can be at most C−1 eigenvectors corresponding to the non-zero eigenvalues. Construct the projection matrix $W=[w_{1},\ldots ,w_{C-1}]$, which will be employed for extracting feature vectors in Equations (6) and (7) [25]:

(6) $$A_{com}=[x_{com}^{1}-\mu_{com},\ldots ,x_{com}^{C}-\mu_{com}]$$

(7) $$\Omega_{i}=W^{T}x_{m}^{i},\;m=1,\ldots ,N,\;i=1,\ldots ,C$$

(8) $$\Omega_{test}=W^{T}x_{test}$$

The feature vectors $\Omega_{i}$ are referred to as discriminative common vectors, and they are utilized for the classification of red jujube. For recognizing a test sample, $x_{test}$, the feature vector of this test sample, is obtained through Equation (7). Subsequently, it is compared with the discriminative common vector $\Omega_{i}$ of each class using the Euclidean distance. The discriminative common vector identified as the closest to $\Omega_{test}$ is utilized to classify the test sample.

### 2.5. Adaboost

In the ever-evolving landscape of machine learning, researchers continually explore various algorithms and techniques to enhance model performance and robustness. Ensemble learning, a powerful paradigm, stands out by combining the outputs of multiple weak learners to achieve significant improvements over individual models. This concept is rooted in the idea of “crowdsourcing”, where aggregating opinions from multiple experts often yields more accurate results than relying on a single expert.

Among the myriad ensemble learning methods, Adaboost (Adaptive Boosting) has emerged as a particularly noteworthy algorithm. Leveraging its unique weight-adjustment mechanism and iterative training process, Adaboost has achieved notable success in practical applications. Inspired by the notion of model fusion, Adaboost integrates multiple relatively weak classifiers to produce a robust ensemble classifier. Our research aimed to delve into the principles, advantages, and applications of the Adaboost algorithm, offering a comprehensive understanding and utilization of this potent machine learning tool.

### 2.6. Adaboost-CLDA

The Adaboost-CLDA algorithm, which is an ensemble learning algorithm that combines the Adaboost algorithm with the CLDA algorithm. In this algorithm, during each iteration of the Adaboost process, the generated training subset is mapped to the feature subspace of CLDA. The weak classifiers are obtained from the nearest-neighbor classifier in the CLDA feature subspace. At this point, the Adaboost algorithm functions as an adaptive feature selection process. Based on the weighted classification errors generated by the weak classifiers in each round, corresponding weights are assigned to the feature projection vectors. Ultimately, a joint feature subspace is constructed through a voting mechanism, forming a strong classifier. The process of Adaboost-CLDA is described as follows:

Input the training set $S=\{(x_{1},y_{1}),\ldots ,(x_{n},y_{n})\}$, where $x_{i}$ is ith sample. $y_{i}$ is the class label $y_{i}\in y=\{1,2,\ldots ,C\}$.

Initialize the weights of all data: $W_{1}(i)=\frac{1}{n},\;i=1,2,\ldots ,n$.

For *t* = 1, …, *T*

(1) Normalize the weights:
  (9) $$P_{t}(i)=\frac{W_{t}(i)}{\sum_{i=1}^{n}W_{t}(i)}$$
(2) CLDA is executed to derive C−1 (where C is the number of classes) optimal feature vectors. These feature vectors serve as the projection space for training data, resulting in the creation of a new training set denoted as $S^{*}$ (C−1 dimensional data set). $S^{*}=\{(x_{1}^{*},y_{1}),\ldots ,(x_{n}^{*},y_{n})\}$, $x_{i}^{*}=W^{T}x_{i}$.
(3) Utilize the nearest-neighbor classifier (NNC) as the weak classifier, supplying NNC with the distribution $P_{t}(i)$. Obtain a hypothesis $h_{t}:x^{*}\to \{1,2,\ldots ,C\}$ in return.
(4) Compute the error of $h_{t}:\varepsilon_{t}=\sum_{i=1}^{N}P_{t}(i)I(y_{i}\neq h_{t}(x_{i}^{*}))$. If $\varepsilon_{t}=0$ or $\varepsilon_{t}>\frac{1}{2}$, then set t=T−1 and terminate the loop.
(5) Set
  (10) $$\alpha_{t}=\frac{1}{2}\ln[(1-\varepsilon_{t})/\varepsilon_{t}]$$
(6) Update the weights:
  (11) $$P_{t+1}(i)=\frac{P_{t}(i)}{Z_{t}}*\left\{\begin{array}{cc} e^{-\alpha_{t}}, & \mathrm{if}\;y_{i}=h_{t}(x_{i}^{*})\\ e^{\alpha_{t}}, & \mathrm{if}\;y_{i}\neq h_{t}(x_{i}^{*}) \end{array}\right.\mathrm{where}\;Z_{t}=\sum_{1}^{n}P_{t}(i)$$
(7) Generate the final hypothesis:
  (12) $$H(x)=\arg\;\underset{y\in Y}{\max}[\sum_{t=1}^{T}\alpha_{t}I(h_{t}(x^{*})=y)]$$

### 2.7. KNN Classifier

KNN is a kind of instance-based learning algorithm commonly utilized for classification and regression problems. Unlike traditional model-based learning algorithms, KNN does not explicitly learn a function for prediction. Instead, it determines the label of a new data point by examining the labels of its closest neighbors within the feature space. The fundamental idea behind KNN is that similar samples in the feature space probably belong to the same class. For a new data point, KNN identifies the K-nearest training samples and makes predictions based on a majority vote for classification problems. In this study, the KNN algorithm was used to construct a classifier for red jujube samples.

### 2.8. Bayes Classifier

The Naive Bayes classifier first learns the prior probabilities of categories and the conditional probabilities of feature values within each category from known training data. These probabilities can be estimated through methods such as frequency statistics. Then, when new samples need to be classified, the classifier utilizes the learned probability model to compute the posterior probabilities for each category and selects the category with the highest posterior probability as the classification result [28,29].

### 2.9. Software

All the algorithms in this article were executed using Matlab 2014b (The MathWorks, Natick, MA, USA).

## 3. Results

### 3.1. Spectral Analysis

The collected NIR spectra of red jujube spanned a wavelength range of 5894.5–11,111 cm^−1^, revealing distinct features, as illustrated in Figure 2a. Two prominent peaks at 8475 cm^−1^ and 6993 cm^−1^ appeared in the NIR spectra. Beyond 7407 cm^−1^, a significant change in absorbance occurred across all red jujube samples, attributed to O-H and water absorption [30]. The absorbance peaked at 6993 cm^−1^, associated with the first and second frequency multiplications of C-H group stretching vibrations, indicating protein-like substances. This peak might be linked to the first-order and second-order double frequency of the O-H group in water [12]. The varying functional group information among red jujube varieties is mostly contained in the NIR spectra.

### 3.2. Spectral Preprocessing

**Figure 3:**
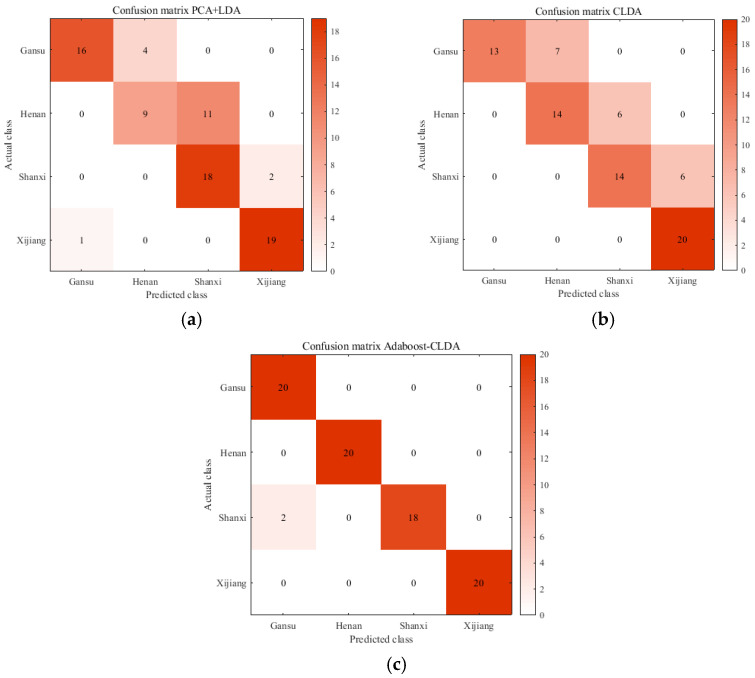
Classification results of PCA + LDA, CLDA, and Adaboost-CLDA. (**a**) The confusion matrix of PCA + LDA; (**b**) the confusion matrix of CLDA; (**c**) the confusion matrix of Adaboost-CLDA.

In this experiment, three preprocessing algorithms denoted as MSC, SNV, and SG filtering, as well as their combinations, were used for spectral preprocessing. Table 1 and Table 2 outline the influence of the three preprocessing algorithms, as well as their combinations, on the classification accuracy with the KNN classifier and Bayes classifier, respectively. Based on Table 1 and Table 2, the classification accuracies of Adaboost-CLDA were higher than those of PCA + LDA and CLDA with several preprocessing algorithms. After comprehensive consideration, SG was chosen as a preprocessing algorithm in this identification system. The NIR spectra processed by the SG algorithm are shown in Figure 2b. Figure 3 shows the classification results of PCA + LDA, CLDA, and Adaboost-CLDA combined with SG preprocessing and the KNN classifier. The confusion matrix in Figure 3 illustrates the test sample numbers correctly classified and wrongly classified. It was evident that Adaboost-CLDA had the highest classification accuracy compared with PCA + LDA and CLDA.

### 3.3. Classification with CLDA

**Figure 4:**
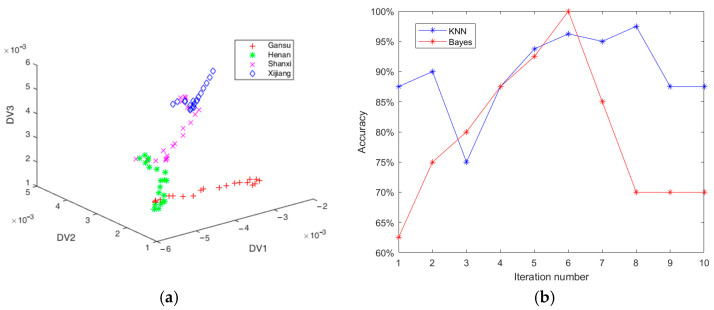
Data distribution and classification. (**a**) The test data projected by three discriminant common vectors of CLDA; (**b**) the classification accuracy of Adaboost-CLDA with KNN and Bayes.

PCA + LDA constitutes a dual-stage algorithm, wherein the data undergo compression through PCA before being extracted by LDA. Different from PCA + LDA, there is no need for CLDA to compress data. CLDA could directly extract discriminant common vectors without the help of PCA. All the 240 red jujube samples were partitioned into the training set (each kind of red jujube has 40 training samples, a total of 160) and the test set (each kind of red jujube has 20 test samples, a total of 80). There are three (i.e., C−1=3) eigenvectors corresponding to the non-zero eigenvalues. That is to say, the projection matrix is three dimensions. CLDA projected the test data into the matrix to obtain the three-dimensional data. Figure 4a shows the test data projected by three discriminant common vectors of CLDA. As illustrated in Figure 4a, certain red jujube samples from Shanxi and Xinjiang could not be differentiated, and a few samples from Gansu and Henan could not be distinguished. Hence, the classification accuracy is only 75%.

### 3.4. Classification with Adaboost-CLDA

The Adaboost-CLDA algorithm, incorporating ensemble learning, achieved optimal experimental results through 10 rounds of iterations. The classification accuracies are shown in Figure 4b. It can be observed from Figure 4b that the highest classification accuracy achieved 97.5% in the eighth iteration with the KNN classifier. From Figure 4b, the highest classification accuracy achieved was 100% in the sixth iteration with the Bayes classifier. The Adaboost-CLDA algorithm had significantly outperformed traditional linear feature extraction methods in terms of classification performance. The reason was that the feature extraction process based on Adaboost autonomously selected the optimal classification features according to the classification errors of weak classifiers. Through the collaborative voting of multiple linear feature extractors, it achieved complex feature extraction from spectral data.

## 4. Discussion

The NIR spectral data were obtained using the NIR-M-R2 spectrometer, and they were preprocessed by SG filtering. Next, PCA + LDA, CLDA, and Adaboost-CLDA were performed for feature extraction from NIR spectra. At last, KNN and Bayes served as classifiers for sample classification. As evident from the experimental results in Table 1, the classification accuracy was influenced by the feature extraction algorithms. When PCA + LDA or CLDA was used, the accuracy dropped below 90%. Conversely, if Adaboost-CLDA served as the feature extraction algorithm, it resulted in an accuracy over 90%. Table 1 indicates that the highest classification accuracy (100%) was attained by combining Adaboost-CLDA and the MSC preprocessing method with the KNN classifier in the identification system for the geographical origin classification of red jujube samples. Table 2 indicates that the highest classification accuracy (100%) was attained by the combination of Adaboost-CLDA and the SG preprocessing method with the Bayes classifier in the classification system.

To consider the effectiveness of the classification system, the number of training and test samples was changed to compute the classification accuracies while keeping other experimental conditions unchanged. Table 3 displays the accuracy of classifying red jujube samples using three feature extraction methods and the numbers of training data and test data. In Table 3, num_training and num_test denote the number of training samples and test samples, respectively. It could be observed that the classification accuracy changed with variation in the parameters num_training and num_test. It was evident that when the parameters num_training and num_test were set to 160 and 80, respectively, the classification accuracy of Adaboost-CLDA achieved the highest value of 97.5%.

The results of this study not only have significance in the classification of the geographical origin of jujubes but also provide a valuable reference for practical market applications. By accurately identifying the geographical origin of jujubes, it is possible to effectively prevent counterfeit products and establish a traceability system for jujube products, safeguarding consumers’ legal rights and interests. Jujubes from different regions have different geographical indications (GI) and prices in the markets. By clarifying the origin information, the study provides a strong basis for geographical origin traceability and better protection of GI.

**Figure 5:**
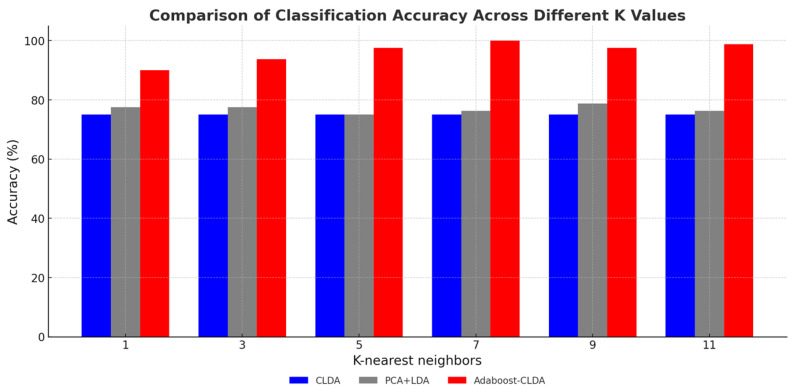
Classification accuracy with different K values using feature extraction methods.

In the study, the role of the parameter K in the K-nearest neighbors (KNN) algorithm was evaluated to understand its impact on classification accuracy. The experimental results demonstrated that the selection of the K value significantly influenced the classification accuracy of KNN combined with feature extraction algorithms, including PCA + LDA, CLDA, and Adaboost-CLDA. As shown in Figure 5, Adaboost-CLDA consistently achieved superior accuracy across various K values, particularly at K = 7, where it outperformed other methods, reaching nearly 100% accuracy. This indicates that the ensemble learning approach of Adaboost-CLDA is robust to the changed value of K and can effectively optimize feature selection during classification. Conversely, CLDA and PCA + LDA displayed lower accuracy rates and greater variability in performance as the K value changed. These findings underscore the importance of selecting an optimal K value in KNN-based classification systems and further affirm the efficacy of Adaboost-CLDA in enhancing classification reliability and robustness.

Recent studies have explored some machine learning and chemometric methods for red jujube classification. Qi et al. (2022) introduced fuzzy improved linear discriminant analysis (FiLDA) to improve the classification of red jujube varieties using NIR spectroscopy [20]. While FiLDA effectively handles noisy data and enhances class separability, its classification accuracy (57.5%) is significantly lower than the accuracy achieved in this study (97.5% with KNN, 100% with Bayes). Furthermore, improved linear discriminant Analysis (iLDA) achieved a low classification accuracy of 76.25%. In contrast, our proposed Adaboost-CLDA approach builds on CLDA’s ability to handle the small sample size problem and further enhances classification accuracy by adaptively selecting optimal features. Unlike FiLDA, which relies on fuzzy membership values, Adaboost iteratively boosts weak classifiers to refine the decision boundary. This makes Adaboost-CLDA a more effective and robust solution for geographical origin classification, as demonstrated by its superior performance with different preprocessing algorithms.

## 5. Conclusions

This study proposes a method for the accurate classification of Chinese red jujube by combining CLDA with Adaboost. While CLDA effectively addresses the “small sample size” problem, the main contribution of our research is the introduction of Adaboost-CLDA, which significantly improves classification accuracy by adaptively selecting features and enhancing the robustness of the classification model. The highest classification accuracy of Adaboost-CLDA could reach 100%. The results indicated that Adaboost-CLDA coupled with near-infrared spectroscopy was an effective method for the geographical origin identification of Chinese red jujube.

The successful application of the Adaboost-CLDA combined with NIR spectroscopy highlights its broad potential in the field of food origin identification and quality control. Beyond red jujube, this method can be extended to other agricultural products such as tea, wine, and rice for geographical indication protection by rapidly analyzing their chemical composition and spectral features, enabling precise classification of origin and variety. For instance, the spectral characteristics of polyphenols and amino acids in tea, the composition of acidity and phenolic compounds in wine, and the starch and protein ratios in rice can all be effectively utilized for high-accuracy origin tracing using this approach. Moreover, this technique can also be applied to honey, coffee, olive oil, and other food products, addressing the growing demand for traceability technologies in the global food market. Its non-invasive, cost-effective, and efficient characteristics offer an environmentally friendly and sustainable solution for the food industry, helping to enhance consumers’ trust, combat counterfeit products, and promote market standardization.

## Figures and Tables

**Table 1:** Classification accuracies of three feature extraction methods with preprocessing algorithms and KNN classifier (%).

Feature Extraction Method	MSC	SNV	SG	MSC + SG	SNV + SG	MSC + SNV
PCA + LDA	53.75	62.5	77.5	78.75	88.75	53.75
CLDA	52.5	47.5	75	67.5	68.75	53.75
Adaboost-CLDA	100	98.75	97.5	98.75	97.5	98.75

MSC, multiplicative scattering correction; SNV, standard normal variable; SG, Savitzky–Golay; PCA, principal component analysis; LDA, linear discriminant analysis; CLDA, common vectors linear discriminant analysis.

**Table 2:** Classification accuracies of three feature extraction methods with preprocessing algorithms and Bayes classifier (%).

Feature Extraction Method	MSC	SNV	SG	MSC + SG	SNV + SG	MSC + SNV
PCA + LDA	71.25	67.5	81.25	78.75	92.5	71.25
CLDA	40	42.5	77.5	50	47.5	40
Adaboost-CLDA	92.5	87.5	100	96.25	91.25	88.75

**Table 3:** Classification accuracies of three feature extraction methods with the numbers of training samples and test samples.

num_training	num_test	PCA + LDA (%)	CLDA (%)	Adaboost-CLDA (%)
120	120	75	54.17	93.34
140	100	82	70	94
144	96	82.29	71.88	96.88
160	80	77.5	75	97.5
180	60	76.67	68.33	90
192	48	72.92	68.75	91.67

## Data Availability

The original contributions presented in this study are included in the article. Further inquiries can be directed to the corresponding authors.
